# Frequency analysis of agricultural drought of maize in Sabie River catchment in South Africa

**DOI:** 10.4102/jamba.v11i1.549

**Published:** 2019-11-06

**Authors:** Eric M. Masereka, George M. Ochieng, Jacques Snyman

**Affiliations:** 1 Department of Civil Engineering, Tshwane University of Technology, Pretoria, South Africa; 2 Department of Civil Engineering, Vaal University of Technology, Vanderbijlpark, South Africa

**Keywords:** empirical frequency analysis, stochastic frequency analysis, root zone, water balance model, agricultural drought

## Abstract

Maize (*Zea mays* L.) is a staple food in South Africa. Under dryland farming, drought is a major limiting factor for maize production. The yield of maize is drastically reduced when rainfall is limited and erratic during the growing season. In order to formulate strategies of reducing the impact of drought on maize production, it is necessary to analyse the magnitude and frequency of drought. The objective of this study was to carry out the magnitude and frequency analysis of agricultural drought events of maize in the Sabie River catchment in order to formulate methods of reducing the impact of drought on maize production in the catchment. The maize growing season in the Sabie River catchment begins in October and ends in February the following year. In this study, the maize growing season was divided into three growing periods based on the month maize is planted. The growing periods were: October to December, November to January and December to February. Simple water balance model in the root zone was applied to determine the minimum amount of rainfall required to meet the water requirement of maize in each growing period in all the eight rainfall zones into which the Sabie River catchment is divided. Empirical frequency analysis and stochastic frequency analysis of the agricultural drought events of maize were carried out. From the study, the return period of agricultural drought events of maize was found to be different for each rainfall zone, and the growing period ranges from 1.78 years to 2.68 years. These results are important for hydrological modellers in that they show that it is necessary to determine the best fit probability distribution for frequency analysis of hydrological events rather than assuming one as the best fit. In all rainfall zones, maize was least prone to drought in the growing period of October to December. Based on the results of the study, development of water resource infrastructure for irrigation and adoption of drought-tolerant varieties of maize was recommended to reduce the high risk of agricultural drought of maize in the Sabie River catchment.

## Introduction

Maize is the staple food for over 70% of the population in South Africa (MIG 2017). But only about 13% of the country is arable and therefore suitable for maize production because of low rainfall and poor soils (MIG 2017). The average annual national production of maize for 5 years up to 2014 was 12.345 million tons per year; however because of drought, there has been a sharp decrease in maize production. In 2015, the production of maize was 10.629 million tons, and in 2016 ending in July, it was 7.597 million tons (FAO 2016). The annual requirement of maize for human and livestock consumption in South Africa is estimated to be 10 million tons per year (Sihlobo & Kapung 2015). The annual national production of maize below 10 million tons per annum leads to food insecurity in South Africa (Mahlangu 2015).

The yield of maize in dryland farming depends mainly among other factors on the amount and distribution of rainfall within the growing season. The yield of maize is drastically reduced when rainfall is limited and erratic within the growing season. Therefore, drought events have a great impact on maize production. The four types of droughts, namely, meteorological, agricultural, hydrological and socio-economical droughts, are caused by deficiency in precipitation. Meteorological drought results when less than normal precipitation is received over a period of time. The long and partially extensive deficiency of precipitation results in hydrological drought, which is a deficiency in bulk water supply that may include low water levels in streams, like reservoir and aquifers (Herm 2002). Socio-economic drought associates drought with supply and demand for economic goods (Edossa, Woyessa & Welderufael 2014). Agricultural drought is exhibited by soil moisture deficit during the growing period of specific crop in specific area. Therefore, agricultural drought for a specific crop in a specific region results when the amount of rain in the growing period does not meet the crop’s seasonal water requirement. From the definition of agricultural drought, drought event of a specific crop in a specific area takes place when the amount and distribution of rainfall in the growing period does not meet the crop’s water requirement.

Generally, yield reduction in most dryland maize growing areas occurs when the seasonal rainfall distribution is erratic (Du Toit et al. 2002). And because rainfall is generally erratic in growing seasons, timing of planting is important to reduce the impact of drought on maize yield. Norwood (2001) indicated that early or late planting can result in lower yields because the probability exists that soil water stress to maize may occur during planting or after planting.

Several studies have been carried out in the field of effects of climate factors on maize yield. Jury (2002) developed maize yield prediction models based on seasonal rainfall. De Jager et al. (1998) developed a framework for forecasting the extent and severity of drought on maize production in Free State Province in South Africa and developed socio-economic and hydrological models to identify maize and wheat production based on climatic data. Most of these studies focused on wide area or regional impact of drought on maize. Literature on the impact of drought on maize yield at tertiary and secondary catchment levels in South Africa is limited. And yet although meteorological drought usually covers vast areas, agricultural drought can be localised because of other localised factors. These factors include planting time within the season, soil properties and seasonal rain characteristics that include onset of rain and rain distribution within the growing season (De Jager et al. 1998). In order to develop strategies of reducing the impact of drought on maize production, it is necessary to carry out frequency analysis of drought.

The main purpose of this study was to carry out frequency analysis of agricultural drought of maize in the Sabie River catchment. Other objectives of the study were:

- to determine the minimum amounts of rainfall required in growing period to meet the water requirement for maize in each of the eight rainfall zones in the Sabie River catchment
- to determine the best fit models for frequency analysis of agricultural drought events of maize in each of the eight rainfall zones in the Sabie River catchment
- to carry out empirical frequency analysis and stochastic frequency analysis of agricultural drought events of maize in growing period and rainfall zones in which maize is least and most prone to agricultural drought in the Sabie River catchment
- to propose methods of reducing agricultural drought of maize risk
- to propose climate change adaptation methods for maize production in the Sabie River catchment.

The crop failure because of drought depends on several factors which include, among other factors, the amount and distribution of soil moisture in the crop root zone throughout the growing period of the crop. The amount and distribution of soil moisture in the crop root zone depends on elements such as amount and distribution of rainfall, the crop evapotranspiration, the runoff and deep percolation during the growing period of the crop. To determine the agricultural drought of a specific crop in a specific area in specific growing period, these elements have to be determined. Several models have been developed to determine these elements. Schulze (1995) adapted the soil conservation service (USDA 1985) model for use in South Africa to estimate runoff at catchment level. The model was defined as:

$$Q={\left(PI-cS\right)}^{2}/(PI+S(1-c))$$ [Eqn 1]

where *Q* = surface runoff depth (mm), *c* = coefficient of initial abstraction and *S* = potential maximum water retention of soil.

Allen et al. (1998) developed a model for evapotranspiration determination from cropped surface using the dual crop coefficient method. The model was defined as:

$$ET_{C}=(K_{cb}+K_{e}).ET_{o}$$ [Eqn 2]

where *ET*_*C*_ = evaporation from a cropped surface (mm), *K*_*cb*_ basal crop coefficient, *K*_*e*_ coefficient controlling evaporation from the soil, *ET*_*o*_ reference evaporation from a hypothetical short-grass surface (mm).

Bennie et al. (1994) reported deep percolation of values ranging from 5% to 20% of rainfall in the growing period under semi-arid sandy soils, but less in clay soils, and upward fluxes of soil water of between 0% and 8% of mean rainfall in a growing period in South Africa. In this study, evapotranspiration, runoff and deep percolation elements were quantified to determine the minimum amount of rainfall required in each growing period in the eight rainfall zones in the Sabie River catchment so that there would be a reduction in maize production because of agricultural drought.

Empirical frequency analysis and stochastic frequency analysis of hydro-meteorological events including agricultural drought events have been carried out in South Africa. Log-Pearson 3 (LP3) probability distribution function has been recommended for the frequency analysis of hydro-meteorological events in South Africa (Alexander 1990, 2001). Gorgens (2007) used both the LP3 and general extreme value (GEV) distribution and found them suitable for frequency analysis of hydro-meteorological events in South Africa. Mkhandi et al. (2000) found that the Pearson Type 3 probability distribution function fitted with probability weighted moments method be the most appropriate distribution to use in frequency analysis of hydro-meteorological events in 12 of the 15 relatively homogenous regions identified in Southern Africa. Recent studies have shown that the best fit models for frequency and magnitude analysis of hydro-meteorological events vary in space and time (Masereka et al. 2016). Therefore, for frequency analysis of hydro-meteorological events, it is advisable to firstly identify and determine the best fit model for frequency analysis of the hydro-meteorological events in question rather than just adopt one. In this study, the method developed by Masereka et al. (2016) was applied to identify the best fit probability distribution functions for frequency analysis of agricultural drought events of maize in each growing period in each of the eight rainfall zones in the Sabie River catchment.

## Material and methods

### Location of the study area

**FIGURE 1:**
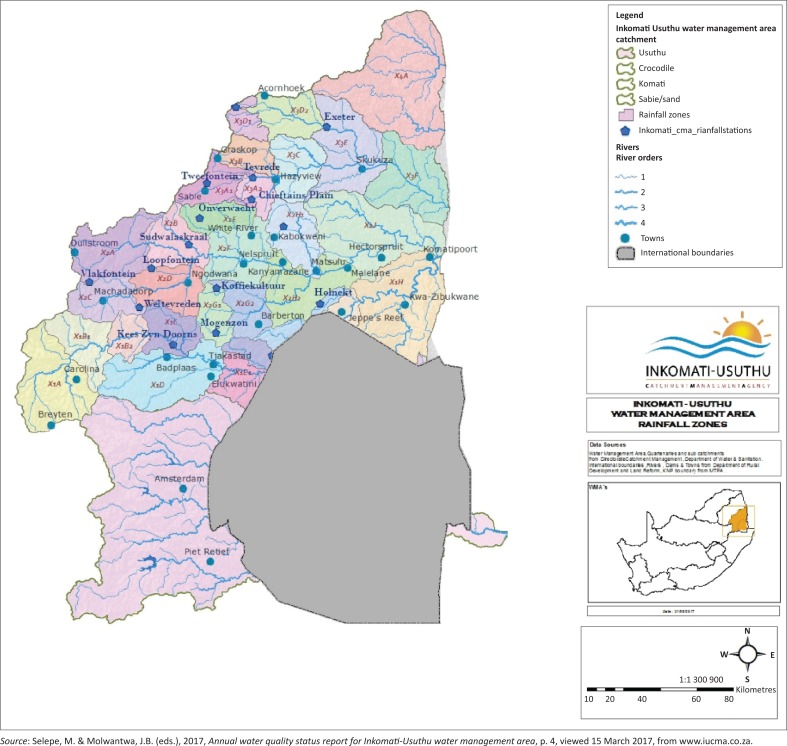
Map showing the Inkomati-Usuthu catchment management area rainfall zones.

The Sabie River catchment forms part of the Inkomati-Usuthu Catchment Management Area (IUCMA) which is allocated in North East South Africa. The area of the Sabie River catchment is 7096 km^2^. The catchment is divided into three tertiary catchments. The three tertiary catchments are divided into 25 quaternary catchments. The 25 quaternary catchments are clustered into eight rainfall zones. The eight rainfall zones are X3A1, X3A2, X3B, X3C, X3D1, X3D2, X3E and X3F. The rainfall zones in IUCMA are shown in Figure 1.

### Data

The maize growing season in all the eight rainfall zones of the Sabie River catchment begins in October and ends in February. In this study, the growing season was divided into three overlapping growing periods. The three overlapping growing periods were October to December, November to January and December to February, depending on the month of planting of maize. The data for the period 1920–2010 were obtained online (Department of Water and Sanitation [DWS] 2017). The data included area of each rainfall zone, monthly mean rainfall, mean monthly evaporation and mean monthly runoff. The area, mean annual evaporation and mean annual precipitation for each rainfall zone are presented in Table 1.

**TABLE 1:** Quaternary catchments and rainfall zones of the Sabie River catchment.

Quaternary catchments	Catchment area (km^2^)		S-pan evaporation		Rainfall	
	Gross	Net	Evap zone	MAE (mm)	Rainfall zone	MAP (mm)
X31A	229.7	229.7	5A	1418.6	X3A1	1218
X31B	198.0	198.0	5A	1425.6	X3A1	1262
X31C	153.5	153.5	5A	1421.8	X3A1	1295
X31D	189.4	189.4	5A	1442.6	X3A2	960
X31E	212.7	212.7	5A	1430.8	X3B	1256
X31F	91.4	91.4	5A	1424.7	X3B	1329
X31G	167.7	167.7	5A	1454.2	X3B	953
X31H	61.3	61.3	5A	1420.8	X3A1	1164
X31J	153.7	153.7	5A	1432.9	X3A2	883
X31K	490.9	490.9	5A	1476.3	X3C	672
X31L	295.0	295.0	5A	1478.0	X3C	735
X31M	713.9	713.9	5A	1483.9	X3E	569
**Tertiary (X31)**	**2957.2**	**2957.2**	**-**	**1456**	**-**	**706**
X32A	110.5	110.5	5A	1440.6	X3D1	1092
X32B	54.4	54.4	5A	1460.4	X3D1	960
X32C	230.7	230.7	5A	1491.0	X3D2	766
X32D	98.0	98.0	5A	1450.0	X3D1	1139
X32E	79.6	79.6	5A	1454.5	X3D1	906
X32F	163.3	163.3	5A	1493.2	X3D2	728
X32G	339.5	339.5	5A	1493.0	X3D2	662
X32H	482.0	482.0	5A	1488.4	X3E	639
X32J	351.3	351.3	5A	1483.4	X3E	593
**Tertiary (X32)**	**1909.2**	**1909.2**	**-**	**1482**	**-**	**730**
X33A	601.6	601.6	5A	1469.8	X3F	540
X33B	316.9	316.9	5A	1461.9	X3F	525
X33C	178.5	178.5	5A	1452.7	X3F	484
X33D	311.1	311.1	5A	1453.5	X3F	469
**Tertiary (X33)**	**1408.1**	**1408.1**	**-**	**1462**	**-**	**514**

*Source*: Middleton, B. & Bailey, A.D., 2005, *Water resources of South Africa. 2005 study (WR 2005)-2011*, viewed 08 June 2017, from www.wrc.org.

Note: Data set in bold indicates the total catchment area, s-pan evaporation and rainfall data for each of the X31, X32 and X32 Tertiary catchments.

MAE, mean annual evaporation; MAP, mean annual precipitation.

### Growing period of maize rainfall data

The total rainfall amounts for each of the three growing periods of maize in each year in all the eight rainfall zones were calculated for the period 1920–2010. The total rainfall amount for each growing period in each rainfall zone was obtained by adding the rainfall amounts of the three months that made the specific growing period in a specific rainfall zone.

### The required minimum amounts of rainfall in the growing period

Several models have been applied to estimate the minimum amount of rainfall required for the optimum growth of crops. The models include macroscopic scale soil moisture dynamic model (Malik, Murty & Narda 1988), soil-water balance model (Driessen 1986), soil-vegetation-atmosphere transfer model (SVAT) (Noilhan & Planton 1989), two-source energy balance (TSEB) model (Norman, Kustas & Humes 1995) and remote sensing surface energy balance algorithms for land (SEBAL) model (Bastiaansen, Menenti & Fedders 1998). In all these models, detailed climatic, soil and crop data were required to develop them. In this study, a simplified root zone water balance model (Driessen 1986) was modified and applied to determine the minimum amount of rainfall required to meet the water requirement of maize in each growing period in each rainfall zone. The elements in this model are presented in Figure 1. The modified water balance in the root zone model for the growing period was defined as:

$$SR_{nm}=Ep_{nm}+Rf_{nm}+Bf_{nm}+Sf_{nm}+If_{nm}$$ [Eqn 3]

where *SR*_*nm*_ was the amount of rainfall required to meet the water requirement of maize in rainfall zone *n* in growing period *m, Ep*_*nm*_ was the evapotranspiration from maize crop in rainfall zone *n* in growing period m, *Rf*_*nm*_ was the runoff from rainfall zone *n* in growing period *m, Bf*_*nm*_ was the base flow from rainfall zone *n* in growing period *m, Sf*_*nm*_ was the subsurface flow from rainfall zone *n* in growing period *m* and *If*_*nm*_ was the interflow from rainfall zone *n* in growing period *m*. Equation 3 was simplified to:

$$SR_{nm}=Ep_{nm}+Rf_{nm}+D_{nm}$$ [Eqn 4]

where *D*_nm_ represents deep percolation which was estimated by:

$$D_{\text{nm}}=Bf_{\text{nm}}+Sf_{\text{nm}}+If_{\text{nm}}$$ [Eqn 5]

### Agricultural drought events of maize

Agricultural drought events of maize in each growing period were identified by peaks below the threshold (PBT) method. In each rainfall zone, the minimum required amount of rainfall (*SR*_*nm*_) to avoid agricultural drought for maize in each growing period was calculated by applying Equation 4. The calculated *SR*_*nm*_ for each growing period in each rainfall zone was taken as a threshold. The rainfall totals in a growing period less than *SR*_*nm*_ were identified as the agricultural drought events for maize in that specific growing period in that specific rainfall zone.

### Statistical analysis

Two types of statistical analysis of agricultural drought events were carried out: empirical statistical analysis and stochastic frequency analysis.

### Empirical statistical analysis

The empirical statistical analysis was applied to determine the return periods of agricultural drought of maize in years up to 90 years. The length of record data was 90 years (1920–2010). Specifically, the method of plotting position (Weibull 1939) was applied to determine the non-exceedance probability *p* and return period *T* of the agricultural drought events in growing periods and rainfall zones in which maize was least and most prone to agricultural drought. The non-exceedance probability (*p*) of the agricultural drought events of maize was determined by the rank-order method. This method involved ordering the events from the smallest event to the largest event. Rank 1 was assigned to the smallest event and rank 90 to the largest event. The data sample size was 90 events.

To obtain the probability (*p*) of each event, Weibull formula (Weibull 1939) was applied:

p=i/n+1 [Eqn 6]

where *p* is the non-exceedance probability for an event with rank *i, i* is the rank of the event and *n* is the sample size which was equal to 90 in this case. The return period (T) of each event is defined as the inverse of its non-exceedance probability (Weibull 1939):

T=1/p [Eqn 7]

The empirical return period in years of each agricultural drought event of maize was determined by applying Equation 7.

In this study, only the return periods of agricultural drought for maize in the growing periods and rainfall zones in which maize was most and least prone to agricultural drought were determined.

### Stochastic frequency analysis

The stochastic frequency analysis involved the identification of best fit models for describing the maize agricultural drought events, determination of the parameters of the identified best fit models and determination of the frequency and magnitude of the agricultural drought events for maize.

The methodology of identifying candidate and best fit models for stochastic frequency analysis of hydro-meteorological extreme events has been comprehensively described (Masereka et al. 2016). The methodology was applied to identify and select best fit models for the frequency and magnitude analysis of maize agricultural drought events in each growing season in each rainfall zone in this study. Only frequency and magnitude analysis of maize agricultural drought events in the growing period and rainfall zone in which maize was most and least prone to agricultural drought was carried out for return periods of 5, 10, 20, 50 and 90 years.

### Quantile-return period models

The quantile-return (Q-T) models based on the identified best fit probability distribution functions for frequency analysis of maize agricultural drought events in the least and most growing season and rainfall zone were developed by incorporating the descriptive statistics of the corresponding data of agricultural drought events (Masereka et al. 2016).

### Confidence intervals of estimated quantiles

The confidence intervals of estimated quantiles of maize agricultural drought events in which maize is least and most prone to agricultural drought were developed by applying the model defined by Raynal-Villasenor (2012):

$$X_{l}=Q_{T}\pm Z_{Z}S_{T}$$ [Eqn 8]

where *X*_1_ = confidence limit, *Q*_*T*_ = design value, *Z*_*X*_ = standard value corresponding to the level of *α* and *S*_*T*_ = standard deviation of estimates.

### Ethical considerations

This article followed all ethical standards for research without direct contact with human or animal subjects.

## Results and discussions

### Rainfall requirements of maize

In the Sabie River catchment, 90% of maize is grown under dryland farming and only 10% is grown under irrigation (DARDLEA 2017). The Sabie River and three farm dams are the main source of irrigation. The farm dams need to be upgraded in order to meet the demand for water for irrigation (DARDLEA 2017).

The results of rainfall requirements for maize in each growing period in the eight rainfall zones in the Sabie River catchment are presented in Table 2. From the results, the rainfall requirement for maize is lowest (456.79 mm) in the growing period of October to December in the rainfall zone X3C and it is highest (627.90 mm) in the growing period of December to February in the rainfall zone X3B. These results show that maize is least prone to agricultural drought in the growing period of October to December in the rainfall zone X3C, and it is most prone to drought in the growing period of December to February in the rainfall zone X3B.

**TABLE 2:** Evapotranspiration, runoff, deep percolation and rainfall requirements of maize in specific growing periods in the eight rainfall zones.

Rainfall zone	Evapotranspiration (mm)	Runoff (mm) in growing period			Deep percolation (mm) in growing period			Rainfall requirements (mm) in growing period		
		October to December	November to January	December to February	October to December	November to January	December to February	October to December	November to January	December to February
X3A1	388.60	160.31	189.67	190.07	26.71	31.61	31.68	575.62	609.88	610.35
X3A2	392.99	110.95	132.76	134.47	26.42	31.60	32.02	530.36	557.35	559.48
X3B	392.67	161.93	197.38	202.71	26.12	31.84	31.69	580.72	621.89	627.90
X3C	403.75	26.52	31.90	32.11	26.52	31.90	32.11	456.79	467.55	467.97
X3D1	396.70	122.82	148.40	151.43	26.70	32.26	32.92	546.22	577.36	581.05
X3D2	407.92	38.00	45.48	45.32	27.15	32.48	32.37	473.07	485.77	485.61
X3E	416.15	35.95	38.99	39.12	26.55	31.65	31.86	478.65	486.79	487.13
X3F	409.61	32.03	38.00	46.36	26.69	31.66	31.14	468.33	469.27	477.11

### Frequency of agricultural drought of maize in the Sabie River catchment

The empirical frequency analysis of agricultural drought events of maize in the growing period of October to December in the rainfall zone X3C and in the growing period of December to February in the rainfall zone X3B is presented in Tables 3 and 4, respectively. Maize is least prone to agricultural drought in the rainfall zone X3C. In this rainfall zone, the empirical return period of agricultural drought is 2.68 years in the growing period of October to December. Maize is most prone to agricultural drought in the rainfall zone X3B. The empirical return period of agricultural drought events of maize in the growing period of December to February in this rainfall zone is 1.78 years.

**TABLE 3:** Rainfall totals, plotting positions and return periods of maize prone to agricultural drought in the growing period of October to December in the rainfall zone X3C.

Rank	X (mm)	Pi	T (years)
1	278.0	0.01	90.00
2	298.1	0.02	45.50
3	300.4	0.03	30.33
4	302.5	0.04	22.75
5	326.6	0.05	18.20
6	333.5	0.07	15.17
7	335.5	0.08	13.00
8	337.4	0.09	11.38
9	339.7	0.10	10.11
10	349.4	0.11	9.10
11	350.8	0.12	8.27
12	355.8	0.13	7.58
13	356.1	0.14	7.00
14	361.4	0.15	6.50
15	362.4	0.16	6.07
16	368.6	0.18	5.69
17	377.6	0.19	5.35
18	382.9	0.2	5.06
19	390.1	0.21	4.79
20	392.2	0.22	4.55
21	392.5	0.23	4.33
22	400.1	0.24	4.14
23	400.4	0.25	3.96
24	419.0	0.26	3.79
25	419.2	0.28	3.64
26	421.5	0.29	3.50
27	425.9	0.30	3.37
28	430.0	0.31	3.25
29	436.08	0.32	3.12
30	440.9	0.33	3.03
31	442.0	0.34	2.94
32	452.2	0.35	2.84
33	454.2	0.36	2.76
34	455.0	0.37	2.68
35	460.7	0.38	2.6
36	462.5	0.4	2.53
37	467.7	0.41	2.46
38	467.7	0.41	2.46
39	478.2	0.43	2.33
40	478.6	0.44	2.28
41	478.8	0.45	2.22
42	498.0	0.46	2.12
43	498.4	0.47	2.07
44	504.7	0.48	2.06
45	505.4	0.49	2.02
46	506.9	0.50	2.00
47	515.8	0.52	1.94
48	517.2	0.53	1.90
49	517.9	0.54	1.86
50	518.1	0.55	1.82
51	520.5	0.56	1.78
52	528.2	0.57	1.75
53	536.5	0.58	1.72
54	537.9	0.59	1.69
55	546.3	0.60	1.65
56	559.1	0.62	1.63
57	561.9	0.63	1.60
58	564.1	0.64	1.57
59	571.3	0.65	1.54
60	573.7	0.66	1.52
61	579.4	0.67	1.49
62	580.6	0.68	1.47
63	589.1	0.69	1.44
64	592.3	0.70	1.42
65	603.9	0.71	1.40
66	612.5	0.73	1.38
67	621.9	0.74	1.36
68	630.7	0.75	1.34
69	631.0	0.76	1.32
70	633.1	0.77	1.30
71	639.8	0.78	1.28
72	650.8	0.79	1.26
73	653.7	0.80	1.25
74	654.6	0.81	1.23
75	669.4	0.82	1.21
76	669.8	0.84	1.12
77	679.8	0.85	1.18
78	687.1	0.86	1.17
79	692.8	0.87	1.15
80	692.9	0.88	1.14
81	700.6	0.89	1.12
82	720.4	0.90	1.11
83	750.7	0.91	1.10
84	753.9	0.92	1.08
85	764.6	0.93	1.07
86	792.8	0.95	1.04
87	838.2	0.96	1.05
88	877.4	0.97	1.03
89	883.0	0.98	1.02
90	901.3	0.99	1.01

X, event; Pi, non-exceedance probability of event i; T, return period.

**TABLE 4:** Rainfall totals, plotting positions and return periods of maize prone to agricultural drought in the growing period of December to February in the rainfall zone X3B.

Rank	X (mm)	Pi	T (years)
1	285.0	0.01	90.00
2	299.2	0.02	45.50
3	303.6	0.03	30.33
4	309.2	0.04	22.75
5	316.0	0.05	18.20
6	324.2	0.07	15.17
7	335.1	0.09	13.00
8	354.3	0.09	11.38
9	402.0	0.10	10.11
10	402.9	0.11	9.10
11	406.9	0.12	8.27
12	413.8	0.13	7.58
13	414.2	0.14	7.00
14	424.3	0.15	6.50
15	426.1	0.16	6.07
16	454.5	0.18	5.69
17	462.2	0.19	5.35
18	471.2	0.20	5.06
19	477.4	0.21	4.79
20	480.8	0.22	4.55
21	492.1	0.23	4.33
22	493.9	0.24	4.14
23	499.5	0.25	3.96
24	501.4	0.26	3.79
25	501.6	0.27	3.64
26	510.2	0.29	3.50
27	512.8	0.30	3.37
28	513.9	0.31	3.25
29	521.7	0.32	3.14
30	525.8	0.33	3.03
31	528.3	0.34	2.94
32	530.2	0.35	2.84
33	532.2	0.36	2.76
34	547.4	0.37	2.68
35	553.2	0.38	2.60
36	553.7	0.4	2.53
37	555.9	0.41	2.46
38	556.0	0.42	2.47
39	559.5	0.43	2.33
40	561.9	0.44	2.26
41	583.2	0.45	2.22
42	585.3	0.46	2.12
43	597.8	0.47	2.07
44	600.7	0.48	2.06
45	603.5	0.49	2.02
46	603.9	0.51	2.00
47	607.8	0.52	1.94
48	608.1	0.53	1.90
49	608.3	0.54	1.86
50	621.5	0.55	1.82
51	624.0	0.56	1.78
52	656.8	0.57	1.75
53	669	0.58	1.72
54	669.8	0.59	1.67
55	670.2	0.60	1.65
56	673.8	0.62	1.63
57	694.9	0.63	1.60
58	695.8	0.64	1.57
59	700.9	0.65	1.54
60	707.8	0.66	1.52
61	731.2	0.67	1.49
62	747.2	0.68	1.47
63	747.3	0.69	1.44
64	748.4	0.70	1.42
65	751.4	0.71	1.40
66	759.6	0.73	1.38
67	767.4	0.74	1.36
68	768.5	0.75	1.34
69	775.9	0.76	1.32
70	781.0	0.77	1.30
71	782.5	0.78	1.28
72	788.3	0.79	1.26
73	799.5	0.80	1.25
74	818.1	0.81	1.23
75	861.0	0.82	1.21
76	865.5	0.83	1.20
77	872.5	0.85	1.18
78	879.3	0.86	1.17
79	945.6	0.87	1.15
80	956.5	0.88	1.14
81	971.6	0.89	1.12
82	982.9	0.90	1.11
83	1007.6	0.91	1.10
84	1045.4	0.92	1.08
85	1073.2	0.93	1.07
86	1090.6	0.95	1.06
87	1194.4	0.96	1.05
88	1380.5	0.97	1.03
89	1413.7	0.98	1.02
90	1456.9	0.99	1.01

X, event; Pi, non-exceedance probability of event i; T, return period.

### Occurrence of agricultural drought of maize at least once in 5 years

The probability of occurrence of agricultural drought of maize at least once in five years was found to be 0.99 in the growing period of December to February in the rainfall zone X3B. The return period of agricultural drought for maize was 1.78 years in this growing period and rainfall zone (Table 5). The probability of occurrence of agricultural drought of maize was found to be 0.95 in the growing period of October to December in the rainfall zone X3C. The return period of agricultural drought for maize was found to be 2.68 years in this growing period and rainfall zone (Table 5). These results confirm the conclusion that maize is most prone to agricultural drought in the growing period of December to February in the rainfall zone X3B in the Sabie River catchment because the probability of occurrence of agricultural drought of maize at least once in five years is the highest and the return period of the agricultural drought of maize is the lowest in all growing periods and rainfall zones.

**TABLE 5:** Rainfall requirements and agricultural return periods.

Variable	Growing periods		
	October to December	November to January	December to February
**X3B**			
Rainfall requirement (mm)	580.72	621.89	627.90
Drought return period (years)	2.22	1.82	1.78
**X3C**			
Rainfall requirement (mm)	456.79	467.55	467.99
Drought return period (years)	2.68	2.46	2.46

### Stochastic analysis of the agricultural drought of maize in the Sabie River catchment

The identified best fit models for the frequency and magnitude analysis of agricultural drought of maize in the Sabie River catchment are presented in Table 6.

**TABLE 6:** Best fit distribution functions for the frequency analysis of agricultural drought events in growing periods.

Rainfall zone	October to December	November to January	December to February
X3A1	GEV	GEV	GEV
X3A2	GEV	GEV	LP3
X3B	GEV	LP3	GEV
X3C	GEV	GL	GEV
X3D1	GEV	GEV	GL
X3D2	LP3	GEV	GEV
X3E	GEV	GL	LP3
X3F	GEV	GL	LP3

GL, generalised logistic; GEV, generalised extreme value; LP3, Log-Pearson 3.

The GEV function was the best fit model for agricultural drought events of maize in 15 out of 24 growing periods, whereas LP3 and generalised logistic (GL) were the best fit model for only 5 out of 24 and 4 out 24 growing periods, respectively. From these results, the candidate probability distribution functions for the stochastic frequency of agricultural drought events in the Sabie River catchment were GEV, LP3 and GL as presented in Table 6.

### Frequency analysis of agricultural drought events of maize in the growing period of December to February in the rainfall zone X3B and in the growing period of October to December in the rainfall zone X3C

The identified best fit model for frequency analysis of agricultural drought events for maize in the growing period of December to February in the rainfall zone X3B was GEV distribution. The Q–T model of GEV was defined (Raynal-Villasenor 2012) as:

$$Q_{\tau }=-X_{o}+\frac{\alpha }{\beta }\left\{1-{\left[-l_{n}\left(\frac{1}{T}\right)\right]}^{\beta }\right\}$$ [Eqn 9]

where *X*_*o*_ was the position factor, *α* was the scale factor and *β* as the shape factor. These parameters were determined by the maximum likelihood estimate method. The determined specific factors of agricultural drought events for maize in the growing period of December to February in the rainfall zone X3B were:

$$X_{o}=414.0\;\alpha =86.69\text{ and }\beta =-0.326$$ [Eqn 10]

Substituting the values into Equation 11, the specific Q–T model for the frequency and magnitude analysis for agricultural drought events for maize in the growing period of December to February in the rainfall zone X3B was:

$$Q_{\tau }=414.0-266.6\left\{1-{\left[-l_{n}\left(\frac{1}{T}\right)\right]}^{-0.326}\right\}$$ [Eqn 11]

Estimated quantiles of agricultural drought events for maize in the growing period of December to February with return periods of 5, 10, 20, 50 and 90 years are presented in Table 7. The estimate quantiles are in close agreement with those estimated based on empirical frequency analysis (Table 4). The results indicate that GEV model fairly represents the frequency of agricultural drought events of maize in the growing period of December to February in the rainfall zone X3B.

**TABLE 7:** Q-T results: X3B agricultural drought events of maize in December to February growing period.

Return period (T) (years)	5	10	20	50	90
Lower limit (*Q*_*T*_) (mm)	439.85	337.89	320.54	305.00	296.44
Estimated (*Q*_*T*_) (mm)	452.31	350.53	333.83	318.30	310.56
Upper limit (*Q*_*T*_) (mm)	464.77	363.17	347.12	331.59	324.68

The identified best fit model for the frequency and magnitude analysis of agricultural drought events of maize in the rainfall zone X3C in the growing period of October to December was GEV distribution.

The Q–T model of GEV was defined (Raynal-Villasenor 2012) as:

$$Q_{\tau }=X_{o}+\frac{\alpha }{\beta }\left\{1-{\left[-l_{n}\left(\frac{1}{T}\right)\right]}^{\beta }\right\}$$ [Eqn 12]

where *X*_*o*_ was the position factor, *α* was the scale factor and *β* was the shape factor. These parameters were estimated using the maximum likelihood estimate method. The determined specific factors of agricultural drought events of maize in the growing period of October to December in the rainfall zone X3C were, in this case, *X*_*o*_ = 347.95 *α* = 58.4 and *β* = − 0.34

Substituting the values into Equation 13, the specific Q–T model for the frequency and magnitude analysis for agricultural drought events for maize in the growing period October to December in the rainfall zone X3 C was:

$$Q_{\tau }=347.95-171.11\left\{1-{\left[-l_{n}\left(\frac{1}{T}\right)\right]}^{-0.34}\right\}$$ [Eqn 13]

Estimated quantiles of agricultural drought events for maize in the growing period of October to December in the rainfall zone X3C of return periods of 5, 10, 20, 50 and 90 years are presented in Table 8. The estimate quantiles are in close agreement with those estimated by the empirical frequency analysis apart from the estimates of quantile of return period of 5 years. This could be because of the fact that GEV is less sensitive to estimating quantiles of low return periods. The results indicate that GEV model fairly represents the frequency of agricultural drought events of maize in the growing period of October to December in the rainfall zone X3C.

**TABLE 8:** Q-T results: X3C agricultural drought of maize events in October to December growing period.

Return period (T) (years)	5	10	20	50	90
Upper limit (*Q*_*T*_) (mm)	330.61	313.86	303.35	293.09	288.54
Estimated (*Q*_*T*_) (mm)	322.29	305.54	294.47	284.21	279.11
Lower limit (*Q*_*T*_) (mm)	313.97	297.22	285.59	275.33	269.68

## Discussion

The results from the study also show that the frequency of agricultural drought events of maize vary between the growing periods within the same growing season. This variation may be because of rainfall patterns within the growing season. There is also variation in the frequency of agricultural drought events of maize between rainfall zones. The may be because of different geographical features of the rainfall zones, for example, elevation and soil conditions that affect runoff, and climatic conditions that affect evapotranspiration.

The results of the study have shown that the frequency of agricultural drought events of maize is best described by three probability distribution functions which are GEV, LP3 and GL. However, 62% of the agricultural drought events of maize in growing periods are best described by GEV distribution. These results are important for hydrological modellers in that they show that it is necessary to determine the best fit probability distribution for the frequency analysis of hydrological events rather than assuming one event within a catchment.

### Proposals of disaster risk reduction of agricultural drought of maize in the Sabie River catchment

**FIGURE 2:**
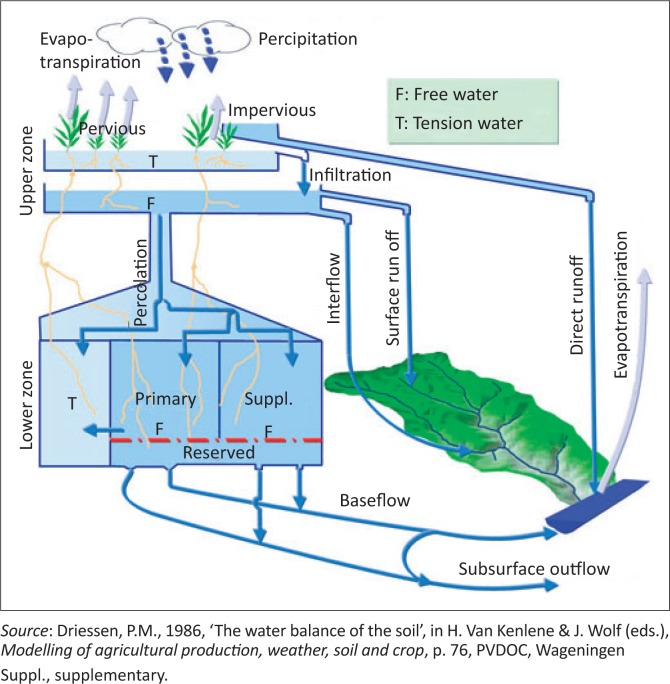
Elements of water balance in root zone model.

From the results of the study (Table 5), the return period of agricultural drought of maize is highest in the growing season of October to December and lowest in the growing period of December to February in the rainfall zones X3C and X3B. This means that early planting of maize in the growing season reduces the chances of failure of maize because of drought. So early planting is one of the recommendations. Other recommendations include increasing soil moisture by better tillage practices, construction of soil and water conservation structures to increase infiltration, reduce runoff and reduce evapotranspiration (minimum tillage) (Figure 2).

### Proposals of climate change adaptation

Based on low quantiles of rainfall expected of return periods 5, 10, 20, 50 and 90 years, it is proposed that more water should be made available for irrigation by renovating and upgrading the existing water infrastructure which includes canals and dams. Also more efficient irrigation systems should be applied. Also drought-tolerant varieties of maize should be adopted.

The results of the study showed that on average, agricultural drought events of maize are experienced in the Sabie River catchment every 1.5–2.5 years. The resilience measure to address this high frequency of agricultural drought of maize has been the introduction of drought-tolerant varieties of maize, which include WE3127, WE 3128, WE4143, WE4144 and WE4145 (MIG 2017). Other resilience measures to reduce the impact of agricultural drought establish irrigation systems for maize production (DARDLEA 2017).

### Practical implications

The results of the study have highlighted the high frequency of agricultural drought events of maize which is the main staple food in the area. This has led to the initiative of renovating and upgrading the ageing water supply and application infrastructure systems in the catchments which include dams, canals and irrigation systems. Also drought-tolerant crops like cowpeas and groundnuts have also been introduced in the catchment (DARDLEA 2017).

### Limitations

The main limitations in this study were short data (90 years) of rainfall amounts in growing periods and limited historical data to model deep flow elements which were inputs in water available in the root zone models for each of the eight rainfall zones.

## Conclusion

The results of the study show that the minimum average total rainfall in a growing period required to reduce the impact of agricultural drought events on maize production is highest (628 mm) in the growing period of December to February in the rainfall zone X3B and lowest (457 mm) in the growing period of October to December in the rainfall zone X3C in the Sabie River catchment.

The results of the study also show that the return period of agricultural drought events of maize varies from growing period to growing period within a growing season. It also varies from rainfall zone to rainfall zone. Frequency of agricultural drought of maize is highest in the growing period of December to February in the rainfall zone X3B with return period of 1.5 years and lowest in the growing period of October to December in the rainfall zone X3C with return period of 2.5 years.

The results of the study show that the candidate probability distribution functions for the frequency analysis of agricultural drought of maize events in the Sabie River catchment were: GEV, LP3 and GL. GEV was the best fit probability distribution function of over 60% of the agricultural drought events of maize.

From the study, empirical frequency analysis and stochastic frequency analysis satisfactorily described agricultural drought events of maize in the Sabie River catchment and can therefore be applied as tools in water use planning for agriculture in this catchment.

From the study, early planting can reduce the impact of agricultural drought of maize in the Sabie River catchment.

As the return period of agricultural drought in the Sabie River catchment is very short (ranging from 1.5 years to 2.5 years), to adapt to climate change, it is necessary to make more water available for irrigation by constructing new dams and renovating and upgrading the water infrastructure in the catchment.
